# Whole blood-derived miRNA profiles as potential new tools for ovarian cancer screening

**DOI:** 10.1038/sj.bjc.6605833

**Published:** 2010-08-03

**Authors:** S F M Häusler, A Keller, P A Chandran, K Ziegler, K Zipp, S Heuer, M Krockenberger, J B Engel, A Hönig, M Scheffler, J Dietl, J Wischhusen

**Affiliations:** 1 Department of Obstetrics and Gynaecology, University of Würzburg, School of Medicine, Josef-Schneider-Strasse 4, Würzburg 97080, Germany; 2 Febit Biomed Gmbh,Im Neuenheimer Feld 519, Heidelberg 69120, Germany; 3 Biomarker Discovery Center Heidelberg, Heidelberg 69120, Germany; 4 Graduate School for Life Sciences, University of Würzburg, School of Medicine, Josef-Schneider-Strasse 4, Würzburg 97080, Germany; 5 Interdisciplinary Center for Clinical Research, University of Würzburg, School of Medicine, Josef-Schneider-Strasse 4, Würzburg 97080, Germany

**Keywords:** ovarian cancer, miRNA profiles, tumour marker screening, monitoring

## Abstract

**Background:**

Screening is an unsolved problem for ovarian cancer (OvCA). As late detection is equivalent to poor prognosis, we analysed whether OvCA patients show diagnostically meaningful microRNA (miRNA) patterns in blood cells.

**Methods:**

Blood-borne whole miRNome profiles from 24 patients with OvCA and 15 age- and sex-matched healthy controls were biostatistically evaluated.

**Results:**

Student's *t*-test revealed 147 significantly deregulated miRNAs before and 4 after Benjamini–Hochberg adjustment. Although these included miRNAs already linked to OvCA (e.g., miR-16, miR-155), others had never before been connected to specific diseases. A bioinformatically calculated miRNA profile allowed for discrimination between blood samples of OvCA patients and healthy controls with an accuracy of >76%. When only cancers of the serous subtype were considered and compared with an extended control group (*n*=39), accuracy, specificity and sensitivity all increased to >85%.

**Conclusion:**

Our proof-of-principle study strengthens the hypothesis that neoplastic diseases generate characteristic miRNA fingerprints in blood cells. Still, the obtained OvCA-associated miRNA pattern is not yet sensitive and specific enough to permit the monitoring of disease progression or even preventive screening. Microarray-based miRNA profiling from peripheral blood could thus be combined with other markers to improve the notoriously difficult but important screening for OvCA.

Patients suffering from ovarian cancer (OvCA) are still burdened by the most unfavourable prognosis of all gynaecological cancers (Pectasides and Pectasides, 2006). This is largely due to the generally late detection of the disease: while 5-year survival is 90% in those 25% of cases in which diagnosis is achieved at FIGO I stage (Duffy *et al*, 2005; Badgwell and Bast, 2007), long-term survival becomes very limited at advanced stages FIGO III and IV (combined 5-year survival rate ∼10%) (Duffy *et al*, 2005). Accordingly, there is a major interest in the discovery of biomarkers for the early detection of OvCA (Clarke-Pearson, 2009). However, even CA125 that was the most promising single marker found in serum is neither sensitive nor specific enough (Meany *et al*, 2009) and therefore not recommended for screening of asymptomatic women (Duffy *et al*, 2005). Sensitivity is biologically limited by the lack of CA125 (over)expression in approximately 50% of OvCAs at FIGO stage I (Jacobs and Bast, 1989). Specificity is also a problem because approximately 1% of all healthy women seem to have elevated levels of this marker (Bast *et al*, 1983). In addition, several benign conditions such as endometriosis, pelvic inflammations, ovarian cysts or even pregnancy (Duffy *et al*, 2005) also result in increased CA125 levels.

As a tool for the monitoring of OvCA recurrence, CA125 is also of very limited use. Until now there is no evidence that an earlier initiation of suitable therapies on increases in CA125 levels translates into a prolonged survival (Eisenhauer *et al*, 1997). Thus, surveillance of OvCA patients with CA125 is not recommended at the moment (Duffy *et al*, 2005).

Considering that all efforts to identify suitable protein biomarkers were largely futile, we turned our attention to microRNAs (miRNAs). These small (17–24 nucleotides) non-coding RNAs (Lee *et al*, 1993) regulate many physio- and pathological processes through control of gene expression (Calin and Croce, 2006; Zhang *et al*, 2007). As opposed to mRNAs, miRNAs are active moieties by themselves and should thus reflect physiological alterations more directly (Gilad *et al*, 2008). A de-regulation of miRNA expression has already been described in numerous malignancies including OvCA in which it was functionally connected to the inhibition of apoptosis (Yang *et al*, 2008; Zhang *et al*, 2008). As tumour-associated miRNA patterns are highly tissue-specific, they can allow an identification of the origin of tumour metastases (Rosenfeld *et al*, 2008). Moreover, miRNAs are also remarkably stable which allows their easy isolation and analysis from tissues and from blood in which they can be found both as free circulating nucleic acids and in mononuclear cells (Chen *et al*, 2008). The possibility to analyse multiple miRNAs in parallel through nucleotide arrays further offers the possibility to increase sensitivity and specificity by using complex miRNA expression patterns as opposed to single biomarkers. Thus, miRNAs might constitute very useful and accessible diagnostic tools (Chen *et al*, 2008; Gilad *et al*, 2008). Accordingly, we used the latest and most complete collection of miRNA sequences analysed to date to identify potential differences between the blood-derived miRNA profiles of OvCA patients and healthy volunteers. On the basis of the findings of our proof-of-principle study, we suggest that this new approach holds considerable promise for the development of improved screening and surveillance strategies for OvCA.

## Materials and methods

### Samples

Blood sampling from OvCA patients and healthy controls has been approved by the local ethics committee. All donors gave written informed consent. The required cohort size was estimated by power analysis. All 24 patients (median age: 64 years, range 29–81 years) had been diagnosed with relapsed OvCA. The time elapsed since the last application of chemotherapy was sufficient to allow for a complete clearance of the drugs. Control samples were obtained from 15 age- and sex-matched volunteers without known disease (median age: 58 years, range 36–83 years). More detailed information on patients is given in Table 1.

### miRNA extraction and microarray screening

Blood samples (5 ml per patient) were collected in PAXgene Blood RNA tubes (BD Biosciences, Heidelberg, Germany) and frozen at −86 °C. After thawing, cellular fractions were obtained by centrifugation (5000 g, 10 min), resuspended in 10 ml RNAse-free water and subjected to total RNA isolation using the miRNeasy kit (Qiagen GmbH, Hilden, Germany). RNA was eluted in water and shipped on dry ice to be analysed on febit́s Geniom real-time analyser (GRTA, febit gmbh, Heidelberg, Germany) using the Geniom Biochip miRNA homo sapiens. Each array contains 7 replicates of 904 miRNAs and miRNA star sequences as annotated in the Sanger miRBase 14.0 (Griffiths-Jones *et al*, 2005, 2008). Samples were biotinylated using either the miRVANA miRNA Labeling Kit (Applied Biosystems Inc., Foster City, CA USA) or by microfluidic-based enzymatic on-chip labeling of miRNAs (MPEA) (Vorwerk *et al*, 2008).

After hybridisation for 16 h at 42 °C, the biochip was washed automatically and a program for signal enhancement was processed with the GRTA. Results were analysed using the Geniom Wizard Software. For each array, the median signal intensity was extracted from the raw data file such that for each miRNA seven intensity values have been calculated corresponding to each replicate copy of miRBase on the array.

After background correction, median values were calculated from the seven replicate intensity values of each miRNA. To normalise arrays, variance stabilising normalisation (VSN) (Huber *et al*, 2002) as implemented in the R package VSN has been applied and all further analyses were carried out using the normalised and background-subtracted intensity values. All microarray data were stored in the freely accessible miRDBXP database (http://64.119.137.93/fmi/iwp), which is designed to store any type of miRNA expression pattern (paper in preparation). In addition, the data have also been deposited in GEO. Additional 24 miRNA profiles from healthy controls – on the basis of a previous version of the Sanger database containing 864 miRNAs – were generously provided by Eckart Meese (University of Homburg, Germany).

### Statistical analysis

The approximate normal distribution of the measured data was verified by Shapiro–Wilk test. Next, miRNAs showing a different behaviour in the two groups were identified by unpaired two-tailed parametric *t*-test. *P*-values obtained for each individual miRNA were adjusted for multiple testing by Benjamini–Hochberg (Hochberg, 1988; Benjamini *et al*, 2001) adjustment. In addition to the single biomarker analysis, samples were also classified according to miRNA patterns as calculated using support vector machines (SVMs, (Vapnik, 1999)) implemented in the R (Team, 2008) e1071 package. In detail, different kernel (linear, polynomial, sigmoid, radial basis function) SVMs were evaluated with the cost parameter being sampled from 0.01 to 10 in decimal powers. The measured miRNA profiles were classified using 100 repetitions of standard 10-fold cross-validation and subsets were selected according to a *t*-test-based filter approach. This means that in each repeat of the cross-validation the *s* miRNAs with lowest *P*-values were computed on the training set Keller *et al*, 2006, with *s* being sampled according to the included number of miRNAs. The respective subset was then used to train the SVM for the prediction of the test samples, which enabled a calculation of the mean accuracy, specificity and sensitivity for each subset size. Permutation tests were applied to check for overtraining. In this study, the class labels were sampled at random and classifications were carried out using the permuted class labels (Keller *et al*, 2006). All statistical analyses were performed using R (Wilcoxon, 1945; R development Core Team, 2008).

## Results

In total, 24 blood samples from patients suffering from relapsed OvCA were analysed. Patient characteristics are provided in Table 1. All patients had already been treated with 1–7 different chemotherapeutic schemes and were about to receive the next chemotherapeutic treatment, none was in palliative-supportive care. However, the time elapsed since the last treatment was >5 half-lives and should thus have been sufficient to allow for a complete metabolic clearance of the administered drugs before the blood draw. Control persons (*n*=15) showed a similar age distribution with a median of 58 years (range from 36 to 83 years) and had no known diseases. Although all OvCA patients have to be considered as post-menopausal after initial debulking surgery, menopausal status was not assessed in the control collective.

**Figure 1:**
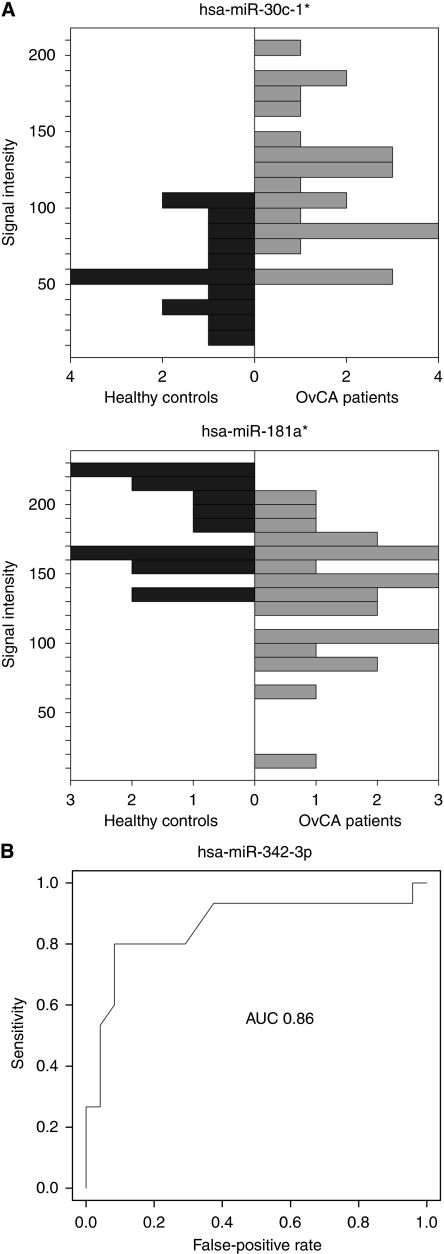
Deregulation of miR-30c-1^*^ and miR-181a^*^ in OvCA samples compared with healthy controls. (**A**) Shown are the intensities of expression for miR-30c-1^*^ (upper panel) and miR-181a^*^ (middle panel) in blood samples from healthy donors (dark grey, *n*=15) or OvCA patients (light grey, *n*=24). *P* values (OvCA *vs* control) were 0.01 for miR-30c-1^*^ and 0.04 for miR-181a^*^, as calculated by Student's unpaired two-tailed parametric *t*-test followed by the Benjamini–Hochberg adjustment for multiple comparisons. (**B**) Receiver operating characteristics (ROC) were generated to show how the sensitivity of OvCA detection and the rate of false positives vary with the discrimination threshold for single miRNAs. Shown is the ROC curve for miR-342-3p. AUC denotes the area under the curve, which is equal to the probability that a classification based on miRNA-342-3p will rank a randomly chosen positive sample higher than a randomly chosen negative one.

Quantitative analysis of miRNAs and miRNA star sequences confirmed a correlation of 0.85 for biological replicates and a variance of 0.005 between these replicates. A comparison between OvCA patients and healthy controls then revealed 147 significantly (*P*<0.05) deregulated miRNAs according to Student's unadjusted *t*-test. After adjustment for multiple comparisons by the Benjamini–Hochberg approach, expression levels of four miRNAs were still significantly different with miR-30c1^*^ being upregulated in OvCA and miR-342-3p, miR-181a^*^ and miR-450b-5p being downregulated. To exemplify these differences, the expression levels of miR-30c1^*^ and miR-181a^*^ are shown in Figure 1A while the receiver operator characteristics (diagram) for miR-342-3p is shown in Figure 1B. Although individual deregulated miRNAs are unlikely to serve as suitable biomarkers for OvCA (as indicated by the highest AUC value of 0.86 for miR-342-3p), they may nevertheless contain some interesting biological information relating to the disease. Thus, the 30 miRNAs that showed the strongest and most consistent deregulations between OvCA patients and healthy controls are provided in Table 2 (in order of increasing *P*-values). Of these, miR-30c-1^*^, miR-191, miR-155, miR-16, miR-106b, miR-146a, miR-29a and miR-383 had already been connected to OvCA as indicated in databases linking miRNAs to specific diseases (www.mir2disease.org and http://cmbi.bjmu.edu.cn/hmdd) (Lu *et al*, 2008; Jiang *et al*, 2009). Moreover, the direction of the observed regulations (up or down) was in perfect agreement with the previously described alterations in OvCA. In total, 15 other miRNAs had, in contrast, never been linked to a specific disease at all. This observation is most likely because of the fact that our study is among the first to rely on miRBase version 14.0 (Griffiths-Jones *et al*, 2008) while most other studies focus on few miRNAs or on significantly smaller sets of older versions. Thus, our screening has revealed some interesting candidate miRNAs that need to be further analysed on a functional level.

To identify characteristic miRNA fingerprints that could be used for classification of the samples as OvCA or controls we used statistical learning techniques, especially SVMs with different kernels, as described above. Classification was performed as recently published (Keller *et al*, 2006, 2009a). 100 iterative repeats of standard 10-fold cross-validation were applied to improve statistical significance. To validate the findings, 100 repetitions were performed with permutated samples that had thus been randomly assigned to either the cancer or the control group in advance.

**Figure 2:**
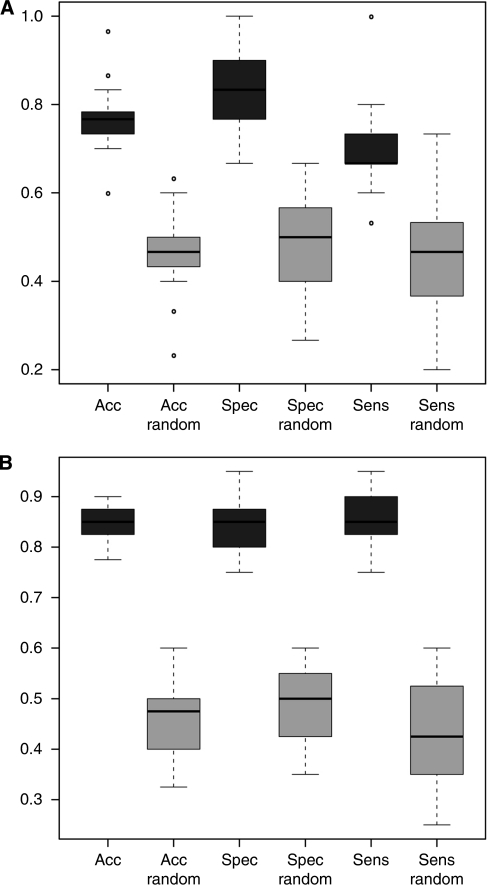
Classification of samples from OvCA patients or healthy controls. (**A**) This classification plot that is based on 60 miRNAs was computed using a radial basis function SVM as described in (Keller *et al*, 2006, 2009a). The black boxes showing the accuracy (‘acc’), specificity (‘spec’) and sensitivity (‘sens’) for classification of all OvCA and control samples (*n*=24 for OvCA, *n*=15 for controls) and were calculated through 100 repetitions of 10-fold cross-validation. The grey boxes show the results obtained when the same mathematical operation is performed in permutation tests (‘random’) in which the class labels (OvCA *vs* control) have been assigned randomly before the values are computed. This is used to validate the classification procedure. The ordinate shows the proportion of samples that were classified correctly to their group. (**B**) Serous OvCA (*n*=20) were compared with an extended group of healthy controls (*n*=39) as in (**A**), using 100 repetitions and 40 miRNAs.

A radial basis function SVM achieved the best result with a subset of the 60 most significantly deregulated miRNAs (see Supplementary Table S1). The classification of the blood samples as ‘OvCA’ respective ‘control group’ was carried out with an accuracy of 76.3 %, a specificity of 83.0% and a sensitivity of 69.7% (Figure 2A). The permutation tests with randomly given class labels showed clearly lower accuracy (40.1%), sensitivity (36.8%) and specificity (41.8%) (Figure 2A), which confirms that the above-mentioned values are not achieved by random guessing. Moreover, when the data from all serous OvCAs (*n*=20) were compared with an extended standard control cohort consisting of individuals without known affection (*n*=39), 40 miRNAs and 100 repetitions sufficed to obtain an accuracy, a specificity and a sensitivity of ⩾85% (Figure 2B), indicating the potential of our approach. As the OvCA group additionally showed miRNA patterns that differ from published profiles for lung cancer (Keller *et al*, 2009a), melanoma (Leidinger *et al*, 2010) or multiple sclerosis (Keller *et al*, 2009b) and from yet unpublished ones for pancreatic cancer, prostate cancer, Wilms’ tumours, pancreatitis, chronic obstructive pulmonary disease, peridontitis, sarkoidosis, heart attacks and unclassified pancreatic diseases (data not shown), our study shows for the first time that OvCA patients show characteristic miRNA signatures in peripheral blood.

## Discussion

In this study, we show that the expression of multiple miRNAs is significantly altered in the blood of OvCA patients as compared with healthy controls. Many of these have never been connected to specific diseases before while eight (miR-30c-1^*^, miR-191, miR-155, miR-16, miR-106b, miR-146a, miR-29a and miR-383) have already been described in the context of OvCA. This may partly be due to the fact that our chip contained many miRNAs that had not been analysed before. Clearly, further studies will be required to elucidate functional consequences of altered miRNA levels in the blood of OvCA patients.

Our study did not follow the current trend to investigate circulating miRNAs in serum from cancer patients (Lodes *et al*, 2009) but rather included the cellular fraction as well. Thus, tumour-specific miRNA profiles contained in exosomes may partly be masked (or, at least, ‘diluted’) by the greater amount of cellular miRNAs. However, experiments performed on lung cancer patients before this study revealed that this is over-compensated by the information contained in the cellular fraction. As the frequency of circulating tumour cells is low in OvCA (median: 15 cells ml^–1^ blood) (He *et al*, 2008), we believe that stromal and myeloid progenitors (Marigo *et al*, 2008; Tu *et al*, 2008) or regulatory T cells, which are recruited to the tumour site (Curiel *et al*, 2004) may significantly contribute to these profiles. Similarly, immunosuppressive and pro-angiogenic signals sent out by the tumour may leave their marks in blood cells. Considering that the formation of a pre-metastatic niche by hematopoietic cells is an early event in tumourigenesis and metastasis (Kaplan *et al*, 2005), it even seems plausible that ‘imprinted’ profiles from blood cells may already be detectable at very early stages of tumour development – whereas miRNAs that are released from cancer cells will only become detectable once a significant neoplastic mass has accumulated.

On the basis of the present data, blood samples from OvCA patients are likely to contain information that can be diagnostically exploited through the combination of microarray-based analysis of complex miRNA fingerprints and statistical learning approaches. Importantly, the profiles obtained for our OvCA collective are clearly distinct from profiles obtained by other centres from patients with lung cancer, prostate cancer, melanoma, pancreatic cancer, Wilms' tumours, pancreatitis, chronic obstructive pulmonary disease, peridontitis, sarkoidosis, heart attacks, multiple sclerosis and unclassified pancreatic diseases (data not shown), which strongly suggests that the observed patterns are indeed disease-specific and not because of nonspecific systemic inflammatory processes or therapy-related toxicity. Thus, a further development of the present approach clearly holds some promise.

At the current stage, however, the predictive power achieved by our proof-of-principle study is too low for a population-wide clinical screening and the values computed for accuracy, specificity and sensitivity could not match those obtained with slightly bigger patient cohorts for lung cancer (Keller *et al*, 2009a) or multiple sclerosis (Keller *et al*, 2009b). Thus, the positive predictive value (PPV), which was 80.4% for our collective would drop to 0.3% if the test was applied to the general population (assuming an annual incidence of 40 OvCA cases in 10^5^ women over 50 years). However, while this is clearly insufficient, preselection of a clinically relevant risk population such as BRCA1 or BRCA2 mutation carriers (Antoniou *et al*, 2003, 2008) could raise the PPV to ⩾10% without further optimisation, simply because of the higher prevalence in this collective. Moreover, the negative predictive value would still be 99.8% for such a collective, which compares fairly well with other markers described for OvCA.

From an economic perspective, miRNA profiling from serum seems rather attractive for clinical diagnostics. Although a wealth of information may be gained for many different conditions, the cost for a single experiment amounts is below US $ 200 (with in-house data generation and analysis). Considering that this allows the measurement of over 900 miRNas in seven replicates, a single data point costs approximately US $ 0.03.

One limitation of this study is certainly the very restricted size of the study collective that makes the results sensitive to individual outliers. In fact, when the miRNA profiles obtained from OvCA patients are compared with the summarised healthy controls from the lung cancer (Keller *et al*, 2009a), the multiple sclerosis (Keller *et al*, 2009b) and our study, accuracy, sensitivity and specificity were significantly increased (data not shown). Biological limitations may further originate from the fact that ovarian carcinomas are very heterogeneous with regard to their histopathological and molecular characteristics (Tinelli *et al*, 2009). Therefore, it might be desirable to establish specific miRNA fingerprints for the individual subtypes of OvCA rather than aiming at a general approach (Köbel *et al*, 2008). This was corroborated by a high variance that we observed within the OvCA group. Consequently, accuracy (87.4%), specificity (88.1) and sensitivity (86.7%) were greatly increased when only serous OvCAs were compared with an extended control group. Still, the investigation of miRNA profiles in a large number of samples from all subtypes of OvCA was clearly beyond the scope of our pilot study.

A second factor that might have added to the variance in both groups was age. Own unpublished observations show that elderly people show significantly altered including miRNA profiles. Consequently, stratification by age groups and menopausal status might improve the distinction between healthy and diseased individuals. Again, this would require a larger study cohort.

For practical reasons, we had chosen patients suffering from a securely diagnosed OvCA as a starting point. However, while the high tumour load in these patients might help the detection of cancer-associated miRNA fingerprints, we could only select treatment-free intervals after platinum-containing therapies for the blood draws. No corrections could be made for the individually adapted treatments the patients had received before their inclusion in our study (compare Table 1), which might introduce a further confounding factor. Considering that all patients had shown progression under previous treatments, the finding by Rui *et al* (2009) that the development of chemoresistance goes along with changes in the miRNA profile of cancer cells might be relevant. Thus, the different therapeutic histories could also contribute to the considerable variance observed in this study. Consequently, stratification according to past treatments might greatly improve sensitivity and specificity for the monitoring of OvCA recurrence through blood-derived miRNA profiles. Alternatively, the link between miRNA fingerprints and chemotherapeutic resistance could also help to find individual and optimal therapeutic regimens for each patient. These issues clearly warrant further investigation, in particular a comparison with treatment-naïve patients (which will be performed in the course of an ongoing study). However, while Rui *et al* described six miRNAs that showed >two-fold alterations on chemotherapy and development of resistance, none of these were among the 30 most significantly deregulated miRNAs in our data set, which strongly suggests that we detected an OvCA-specific rather than a treatment-induced miRNA fingerprint.

Obviously, numerous questions are not answered by our proof-of-principle study and remain to be addressed in follow-on studies. However, our first data already suggest that OvCA samples are associated with characteristic miRNA patterns in peripheral blood. Compared with investigated markers such as CA125, which is neither sensitive nor specific enough for OvCA screening (Duffy *et al*, 2005) or monitoring (Eisenhauer *et al*, 1997), miRNA profiling from peripheral blood seems promising, especially because many possibilities for optimisation remain. Moreover, this new non-invasive strategy could easily be combined with the best current options like the combination of the serum biomarker CA125 with transvaginal ultrasound (Menon *et al*, 2009) or serial determinations of CA125-levels in peripheral blood (Skates *et al*, 2003), which on their own are still not good enough to be recommended for use in clinical routine (Mutch, 2009; Partridge *et al*, 2009). Thus, we believe that the detection of OvCA-associated miRNAs from peripheral blood can become a valuable addition to the current insufficient panel of biomarkers.

## Figures and Tables

**Table 1:** Patient characteristics

**Number**	**Year of birth**	**Histology**	**Current therapy**	**Pre-therapies**	**Tumour load**
1	1980	Serous	Pegylated liposomal doxorubicin	Paclitaxel/carboplatin	++
2	1938	Serous	Topotecan	Paclitaxel/carboplatin; carboplatin mono	++
3	1951	Serous	Gemcitabine	Paclitaxel/carboplatin; pegylated liposomal doxorubicin; topotecan, treosulfan	++
4	1931	Serous	Topotecan	Carboplatin mono	+
5	1937	Serous OvCA or uterine carcinoma	Treosulfan	Paclitaxel/carboplatin; epirubicin; carboplatin mono	+
6	1937	Solid	Topotecan	Cyclophosphamid/carboplatin; carboplatin mono Paclitaxel/carboplatin	+
7	1947	Serous	Topotecan	Paclitaxel/carboplatin	++
8	1939	Serous	Topotecan	Paclitaxel/carboplatin	+++
9	1955	Serous	Pegylated liposomal doxorubicin	Paclitaxel/carboplatin; topotecan	+
10	1931	Serous	Paclitaxel	Carboplatin mono; topotecan; pegylated liposomal doxorubicin; treosulfan	+
11	1954	Serous	Treosulfan	Paclitaxel/carboplatin; topotecan; carboplatin mono; pegylated liposomal doxorubicin	+++
12	1954	Serous	Topotecan	Paclitaxel/carboplatin; pegylated liposomal doxorubicin; HIPEC with mitomycin	++
13	1943	Serous	Gemcitabine	Paclitaxel/carboplatin; peg.-lip. doxorubicin; topotecan; vinorelbin; treosulfan; carboplatin mono; paclitaxel	++
14	1960	Serous	Carboplatin mono	Paclitaxel/carboplatin; topotecan	+
15	1929	Endmetrioid	Carboplatin mono	Carboplatin mono; topotecan; pegylated liposomal doxorubicin; treosulfan	+
16	1963	Endometrioid	Paclitaxel/carboplatin	Carboplatin mono; pegylated liposomal doxorubicin; treosulfan	+
17	1929	Serous	Treosulfan	Carboplatin mono	+
18	1940	Serous	Topotecan	Carboplatin mono; paclitaxel/carboplatin; pegylated liposomal doxorubicin	+++
19	1955	Serous	Pegylated liposomal doxorubicin	Paclitaxel/carboplatin; topotecan; carboplatin mono	+++
20	1950	Serous	Paclitaxel	Paclitaxel/carboplatin	++
21	1946	Serous	Topotecan	Carboplatin mono; pegylated liposomal carboplatin;	+
22	1965	Serous	Paclitaxel/carboplatin	None	+
23	1963	Serous	Paclitaxel/carboplatin	Paclitaxel/carboplatin	++
24	1933	Serous	Pegylated liposomal doxorobicin	Paclitaxel/carboplatin	+

Abbreviations: HIPEC=hyperthermic intraoperative peritoneal chemotherapy; OVCA=ovarian cancer. Tumour load: ‘+’: modest/ ‘++’: high/ ‘+++’: huge.

**Table 2:**
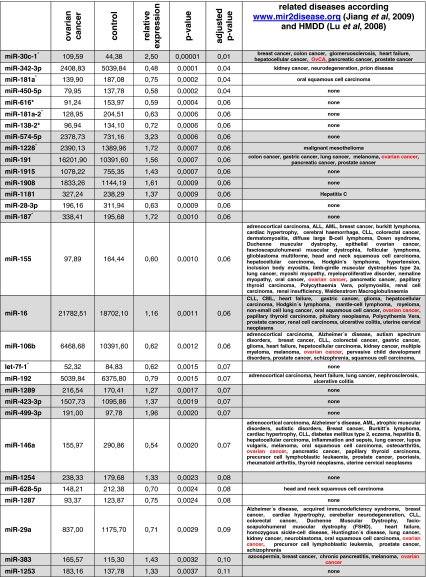
miRNAs showing differential expression between OvCA samples and negative controls
